# Recent Improvements in the Operative Surgery of the Eye

**Published:** 1869-02

**Authors:** 


### Recent Improvements in the Operative Surgery of the Eye.

At the Liverpool Medical Association, October 22d, Mr. T. Bickerton spoke of the recent large advances in this department, and to the advantages offered to the scientific physiologist and pathologist by the peculiar construction of the eye, and by the aid of the opthalmoscope. He drew attention to the treatment of affections of the lacrymal passages, by slitting the puncta lacrymalia on the lower lid, as far as the opening into the sac; and exhibited the instrument used for doing this, and the probes used by Mr. Bowman and Von Graefe. He then showed the ball and needle used for destroying næva materni on the lids. In trichiasis, the treatment recommended by Dr. Williams, of Cork, was, to dip a needle into deliquesced caustic potash, and insert it into each affected hair-bulb, drawing out the hairs three or four days afterward. This should be done early. If the whole row were involved, it was advisable to excise a piece of the skin and cartilage above the edge of the lid, and then bring the parts together by means of sutures, treating supernumerary hairs by caustic potash. In entropium, the present operation is to make two incisions through the skin and tarsi, and part of the orbicularis palpebrarum; to seize the end of the enclosed skin, and, by vertical incisions, to cut through the muscle and remove it entirely, and then to apply sutures, but no dressing. Squint should be operated upon early. Mr. Bickerton described Leibreich's mode of cutting subcutaneously, and the method which he adopted himself, and which he had found exceedingly satisfactory. The instruments used in these operations were exhibited. Excision of the eyeball was devised by O'Farrell, of Dublin, and M. Bonnet, about the same time, and independently of each other. It was first performed by Doctor Storber in 1841; and Mr. Critchett first performed it in this country. The operation was fully described. The hemorrhage, as a rule, was slight, and could be controlled by a stream of water from sponges. A light pad of lint and bandage over the lids was all the dressing required. Abscission of the anterior half of the eye was an operation in which Mr. Bickerton had no faith. He described the modes of operating for soft cataract; 1, by making a small incision into the cornea and evacuating the lens by one or two operations; 2, extraction by suction. The lens may be removed at once by this process; but no more suction-force should be employed than is sufficient to bring it away with ease. The author found that, when the lens was very soft, suction was not required. In hard traumatic cataracts, Von Graef originated the traction operation; and it has been extended to other cataracts by Dr. Schuft. The various steps of the process were explained, and the scoops, etc., shown. This did not give good results; and its place had been taken by Von Graef's modified linear extraction. The author went very fully into the mode of doing this; explaining the various steps and the precautions to be taken, as well as the after-treatment. He had found the results in his own hands more satisfactory than those of any other operation on the eye. Capsular cataract was removed by one or two needles; and, if this failed, by a broad needle through an opening in the cornea. In some cases, it was necessary to enlarge this, and to take away a portion of the iris. Artificial pupil should be made as central and well defined as possible directly behind a clear cornea, and that which was least altered in curvature. The modes of doing it were: 1, removal of a portion of the iris; 2, withdrawing a portion of the iris, iridodesis; 3, incision of the iris. It was required for inflammatory adhesions; dense nebulæ of the cornea over the center of the pupil. In the latter, iridodesis was very useful. The author described the several operations, and also a modified one by himself. He then entered into the question of iridectomy, and spoke strongly in its favor, enumerating the several affections in which it should be employed. The *rationale* of its action was uncertain; but it affected a change in the circulation and in the secretion of aqueous humor. There was a very intimate relation, through the ciliary muscle, between the iris, choroid, and retina. In describing the operation the author said he seldom found it necessary to give chloroform; and that he used Von Graef's narrow knife for modified linear extraction, making the incision on a small scale. He had also done the operation with the conjunctival flap, as in modified linear extraction, and had found it very satisfactory. In considering the relative practical value of iridectomy, division of the ciliary ligament, and division of the ciliary muscle, he was of opinion that each was of very great value in properly selected cases. He then gave an interesting history of the operations for puncturing the eyeball, from the time when it was first practiced by Dr. Whyte, of Manchester, in 1802, up to the present time. In conclusion, he alluded to the evacuation of effusion between the retina, choroid, and sclerotic, by two needles, and laceration of these tissues, as practiced by Messrs. BOWMAN and HULKE.—*Medical and Surgical Reporter.*

